# Effect of WenXin KeLi on Improvement of Arrhythmia after Myocardial Infarction by Intervening PI3K-AKT-mTOR Autophagy Pathway

**DOI:** 10.1155/2022/2022970

**Published:** 2022-09-29

**Authors:** Meng Lv, Ding Yang, Xiaodi Ji, Lixia Lou, Bo Nie, Jiuli Zhao, Aiming Wu, Mingjing Zhao

**Affiliations:** Dongzhimen Hospital Affiliated to Beijing University of Chinese Medicine, Key Laboratory of Chinese Internal Medicine of Ministry of Education and Beijing, Beijing 100700, China

## Abstract

**Background:**

Myocardial infarction (MI) is an acute and serious cardiovascular disease. Arrhythmia after MI can lead to sudden cardiac death, which seriously affects the survival outcome of patients. WenXin KeLi is a Chinese patent medicine for the treatment of arrhythmia in a clinic, which can significantly improve symptoms of palpitation and play an important role in reducing the risk of arrhythmia after MI. In this study, we aimed to explore the pharmacological mechanism of WenXin KeLi in protecting the heart.

**Methods:**

The MI model was established by ligating the left coronary artery and the ventricular fibrillation threshold (VFT) was measured by electrical stimulation. The expression of connexin43 (CX43) and autophagy-related protein were measured by Western Blot, and correlation analysis was conducted to study the relationship between cardiac autophagy, CX43, and arrhythmia in rats after MI. The effects of WenXin KeLi on arrhythmia, cardiac structure, and function in MI rats were respectively observed by electrical stimulation, cardiac gross section, Masson staining, and cardiac ultrasound. The effects of WenXin KeLi on the expression of phosphoinositide 3 kinase-protein kinase B-mammalian targets of rapamycin (PI3K-AKT-mTOR) autophagy pathway and CX43 were observed by Western Blot.

**Results:**

After 4 weeks of MI, the VFT in the model group was significantly reduced, the expression levels of yeast ATG6 homolog (Beclin1), microtubule-associated protein 1A/1B-light chain 3 (LC3II/LC3I), and p-CX43 (S368) significantly increased, the expression of sequestosome-1(P62) and CX43 significantly decreased. LC3II/LC3I and Beclin1 expression were significantly negatively correlated with the VFT, and the expression of P62 and CX43 were significantly positively correlated with the VFT. LC3II/LC3I and Beclin1 expression were negatively correlated with CX43 expression, while P62 expression was positively correlated with CX43 expression. WenXin KeLi could significantly increase the VFT, reduce the deposition of collagen fibers, and increase the index levels of the left ventricular end-diastolic anterior wall (LVEDAW), interventricular septum end-diastolic (IVSED), left ventricular end-systolic anterior wall (LVESAW), interventricular septum end-systolic (IVSES), left ventricular end-diastolic posterior wall (LVEDPW), left ventricular end-systolic posterior wall (LVESPW), left ventricular ejection fraction (LVEF) and left ventricular fractional shortening (LVFS), and reduce the index levels of the left ventricular end-diastolic dimension (LVEDD), left ventricular end-systolic dimension (LVESD), left ventricular end-diastolic volume (LVEDV) and left ventricular end-systolic volume (LVESV). WenXin KeLi could increase the expression of CX43, P62, AKT, p-PI3K, p-AKT (308), p-AKT (473), and p-mTOR and decrease the expression of LC3II/LC3I and Beclin1.

**Conclusion:**

WenXin KeLi can activate the PI3K-AKT-mTOR signaling pathway, improve cardiac autophagy and Cx43 expression in rats after MI, reduce the risk of arrhythmia after MI, and play a cardioprotective role.

## 1. Introduction

Epidemiological reports show that the prevalence of cardiovascular diseases in China is on the rise, cardiovascular disease is the leading cause of death in urban and rural residents [1]. Myocardial infarction (MI) is a serious type of cardiovascular disease. Cardiac structure and function after MI pathological changes and secondary arrhythmia complications, can lead to sudden cardiac death, and influence the outcomes in patients with long-term indicators [2]. Therefore, the study of the internal mechanism of arrhythmia events after MI is the key to improve the prognosis of MI. In recent years, autophagy has attracted much attention in cardiovascular research. Cardiac autophagy can be used not only as a protective factor for myocardial survival but also as a risk factor for cardiomyocyte death [3, 4], affecting the occurrence and development of diseases. The changes in autophagy after MI and its potential influence on complicated arrhythmias are worth studying. Connexin43 (CX43) is the most basic structural protein that constitutes the gap junction in the heart. The decreased expression of CX43 will directly lead to the electrical conduction disorder between myocardial cells [5]. Autophagy is one of the main pathways of protein degradation, and excessive activation of autophagy will accelerate CX43 degradation and even lead to myocardial cell death [6]. Therefore, changes in autophagy level and CX43 expression may be associated with arrhythmia after MI, but it still a lack of experimental verification.

Many evidence-based clinical studies have shown that Traditional Chinese Medicine (TCM) plays a role in improving ischemic diseases. WenXin KeLi is a Chinese herbal compound and is mainly composed of *Codonopsis pilosula*, *Polygonatum kingianum*, *Panax notoginseng*, *Amber*, and *Nardostachy jatamansi* (the percentage is 15 : 20 : 3:2 : 10), which is the first antiarrhythmic Chinese medicine to be approved by the Chinese state. Results of systematic pharmacological studies on WenXin KeLi extract showed that it reduced malignant arrhythmias and shortened RR, PR, and QT intervals [7]. Systematic review and meta-analysis results have proved that WenXin KeLi can effectively reduce the occurrence of complications after MI, play a heart-protective role and prolong the survival period of patients [8, 9]. Whether its mechanism of protecting the heart and reducing the risk of arrhythmia after MI is related to the intervention of cardiac autophagy and improvement of gap junction protein expression is a scientific question worthy of further study. The PI3K-AKT-mTOR pathway is a widely studied autophagy pathway [10]. Our study intends to explore the changes in cardiac autophagy, myocardial CX43 expression levels, and their correlation with arrhythmia in MI rats. On this basis, the cardiac protection mechanism of WenXin KeLi improving CX43 expression and reducing the risk of arrhythmia after MI was further analyzed from the perspective of the PI3K-AKT-mTOR autophagy pathway, hoping to provide new experimental evidence for revealing the molecular mechanism of TCM in the prevention and treatment of arrhythmia after MI.

## 2. Materials and Methods

### 2.1. Animal Model

Male Sprague-Dawley rats (body mass 200g ± 20 g) were purchased from Beijing Weitong Lihua Laboratory Technology Co., Ltd. (license number SCXK2016-0006), and raised in the Environmental Animal Barrier Room of the Key Laboratory of the Ministry of Internal Medicine of Traditional Chinese Medicine. All animal experiments were approved by the Experimental Animal Ethics Committee of Dongzhimen Hospital. According to reference [11], the rat model of myocardial infarction was established by ligation of the anterior descending branch of the left coronary artery. Immediately after ligation, ischemia and whitening of the anterior wall of the left ventricle were observed, the ST segment was significantly elevated, and the pathological *Q* wave was observed 24 hours after the operation.

### 2.2. Group Administration

The pathological *Q* waves with postoperative 24 hours in the anterior wall V3–V6, lateral wall I, AVL lead, and the anteroseptal wall V1 or V2 were used as grouping criteria [12]. The animals were randomly divided into the model group, the metoprolol group, the low dose of WenXin KeLi (WXKL-LD) group, and the high dose of WenXin KeLi (WXKL-HD) group. The sham group was only threaded without ligation. Each group had 8 rats. The doses of WenXin KeLi and the metoprolol group were converted according to the equivalent dose conversion method of reference [13]. WenXin KeLi (5 g per capsule) was provided by Buchang Pharmaceutical Co., Ltd. (Shandong, China), and metoprolol Tartrate tablets (25 mg per tablet) were provided by AstraZeneca Pharmaceutical Co., Ltd. (Jiangsu, China). According to the group, all rats were treated via intragastric administration (1.35 g/Kg/d in the WXKL-LD group, 2.25 × 10^−3^ g/Kg/d in the Metoprolol group, 2.7 g/Kg/d in the WXKL-HD group). Intragastric administration was started 24 hours after the operation, once a day, for 4 weeks. The sham group and model group were given normal saline.

### 2.3. Cardiac ultrasound

Four weeks after the operation, the rats were anesthetized by intraperitoneal injection, and the skin was prepared in a large area of the chest and abdomen. The rat was fixed on the ultrasound test table, and the M-mode curve imaging was performed at the left ventricle beside the sternum guided by two-dimensional ultrasound (small animal ultrasound imaging system Vevo2100, Toronto, Canada). Left ventricular end-diastolic anterior wall (LVEDAW), interventricular septum end-diastolic (IVSED), left ventricular end-diastolic posterior wall (LVEDPW), left ventricular end-diastolic dimension (LVEDD), left ventricular end-diastolic volume (LVEDV), left ventricular end-systolic anterior wall (LVESAW), interventricular septum end-systolic (IVSES), left ventricular end-systolic posterior wall (LVESPW), left ventricular end-systolic dimension (LVESD), left ventricular end-systolic volume (LVESV), left ventricular ejection fraction (LVEF), and left ventricular fractional shortening (LVFS) were measured. At least three consecutive cardiac cycles were selected for all the original measurements and the average values were taken.

### 2.4. Cardiac macroscopic

Four weeks after the operation, the rats were anesthetized. The chest cavity was opened, and the heart was taken out and placed into precooled 4°C normal saline to wash away the residual blood. The heart was placed on a low-temperature container, sterilized gauze was used to absorb the liquid, and a general picture of the heart was taken. The left atrial appendage, right atrium, and right ventricle were cut off by ophthalmic scissors, and the tissue blocks of 4 mm were cut at the largest cross-section of the heart and soaked in a 4% tissue fixative solution. After fixation for 24 hours, the tissue was treated with a fully automatic closed tissue dehydrator and then embedded in paraffin to make a paraffin section of 4 *μ*m thickness.

### 2.5. Masson staining

Paraffin sections of rat myocardial tissue were put into xylene first, then put in the gradient alcohol. Masson staining was performed according to the instructions of the staining kit (D026-1, Nanjing, China). The sections were stained with nuclear dye solution for 60 seconds, rinsed with flushing solution, stained with pulp dye solution for 50 seconds, rinsed with flushing solution, separated with color separation solution for 8 minutes, and directly stained with a redyeing solution for 5 minutes, finally washed with anhydrous alcohol, dried in the ventilation place and sealed. The collagen volume fraction (CVF) was analyzed by Image J software. 8 sections were selected from each group, and 3 nonoverlapping visual fields were collected from each slice for statistical analysis.

### 2.6. The ventricular Fibrillation Threshold (VFT) Detecting

The rats were anesthetized by intraperitoneal injection. Endotracheal intubation was performed, the ventilator was connected to assist respiration, and the biological function system (BL-420S, Chengdu, China) was connected. Then start the system software, select the menu “Input signal/Channel 1/ECG,” and the ECG waveform will appear. Open the rat chest with scissors along the fourth and fifth intercostal lines and expose the heart. The positive pole of the stimulation electrode was hooked at the apex of the heart, and the negative pole was hooked at a distance of 3 mm away from the positive pole near the bottom of the heart. The electrical stimulation conditions (crude voltage, string stimulation, string length of 10 stimulation waves, initial intensity 1V) were set. After the pattern was stabilized, electrical stimulation was started, the condition of ventricular fibrillation induced by electrical stimulation within 15V was recorded, and the voltage value of ventricular fibrillation induced for the first time was taken as the threshold value of ventricular fibrillation.

### 2.7. Immunofluorescence detecting

The paraffin sections were placed on a 60°C slide drier for 1 hour (HI1220, LEICA, Germany), then put into xylene I, xylene II, xylene III for 15 minutes each, anhydrous ethanol I, anhydrous ethanol II, 95% ethanol I, 95% ethanol II, 90% ethanol, 80% ethanol and 70% ethanol for 5 minutes each, and rinsed with PBS buffer 3 times for 5 minutes each. Put the sections into the incubator box containing sodium citrate antigen repair solution and 98°C heat preservation for 20 minutes. After natural cooling, sections were rinsed with PBS buffer 3 times for 5 minutes each, put into 0.3% PBST for 20 minutes, and rinsed with PBS buffer 3 times 5 minutes each. Then rat heart tissue on the section was added to 100 *μ*L 8% donkey serum sealing solution and incubated at 37°C for 30 minutes. Without washing, the CX43 antibody (Abcam company, product number: ab11370) diluted by 5% donkey serum was added, overnight at 4°C. Avoid light operation, add fluorescent secondary antibody on the tissue, incubate at 37°C for 1 hour, wash with PBS buffer solution 3 times, each time for 5 minutes, and add DAPI sealing agent. Images were collected by fluorescence microscope to observe the expression changes of CX43 proteins in each group.

### 2.8. Western Blot Detecting

About 20 mg of tissue was removed from the marginal area of ventricular infarction of the rat, and adding 200 *μ*L of RIPA lysate according to the proportion of 1 : 10 with fully ultrasonic homogenate. After standing for 20 minutes, the supernatant was removed by centrifugation at a low temperature. Protein concentration was determined using a BCA kit, adjusted to 4 *μ*g/*μ*L, and stored at −20°C. SDS-PAGE gel electrophoresis was performed with a sample loading volume of 10 *μ*L per group and a constant flow of 200mAn. The proteins were transferred to the PVDF membrane and sealed at room temperature for 1 hour. After washing membranes with TBST, the diluted primary antibody was added and incubated at 4°C overnight. The dilution ratios of GAPDH, CX43, LC3, P62, and Beclin1 antibodies were 1 : 20000, 1 : 8000, 1 : 1500, 1 : 5000, and 1 : 5000, respectively. The secondary antibody was incubated at room temperature for 1 hour, and the protein band can be visualized by chemiluminescene. ImageJ was used to analyze the protein band and calculate the relative expression levels of each group of proteins.

### 2.9. Confocal detecting

The staining procedure of the paraffin section was the same as that of experiment 7. The mixture of LC3 antibody and CX43 antibody was dropped for each section, and the dilution ratio of LC3 and CX43 were 1 : 300 and 1 : 300 respectively. After staining, images were collected in a darkroom under a confocal laser scanning microscope (TSC SP8 X, LEICA, Germany). The average fluorescence intensity of each protein expression was analyzed by Image J.

### 2.10. Statistical Analysis

SPSS software was used for statistical analysis, and the measurement data were expressed by means ± standard deviation. First, a normality test was carried out. One-way ANOVA was used for normal variables. When the variables were normal and homogeneous, the LSD method was used for inter-group comparison; Dunnett's T3 method was used when variance was uneven, and the nonparametric rank sum test was used when non-normal. Pearson correlation analysis was used for the correlation test. *P* < 0.05 was considered statistically significant.

## 3. Results

### 3.1. Study on the Correlation between Cardiac Autophagy and Arrhythmia in Rats with MI

**Figure 1:**
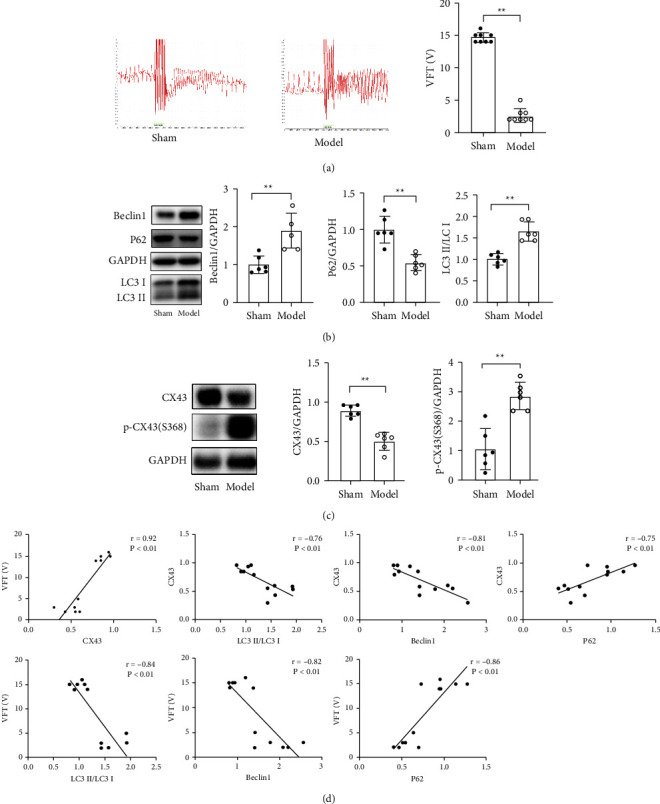
Study on the correlation between cardiac autophagy and arrhythmia in rats with MI: (a) VFT of rats in sham group and model group was measured by electrical stimulation; (b) the expression of autophagy-related proteins in sham group and model group was detected by Western Blot; (c) Western Blot was used to detect CX43 protein expression in sham group and model group; (d) correlation analysis between VFT, CX43, and autophagy in MI rats. Compared with the sham model, ^*∗∗*^*P* < 0.01.

Four weeks after the operation, the VFT in the model group was significantly lower than that in the sham group (Figure 1(a)). Western Blot results showed that the protein expression of Beclin1 and LC3II/LC3I significantly increased and the expression of P62 protein significantly decreased in the model group (Figure 1(b)). In the model group, the expression of CX43 protein decreased significantly, while the expression of p-CX43 (S368) protein increased significantly (Figure 1(c)). Correlation analysis showed that there was a significant positive correlation between CX43 protein expression and the VFT (*r* = 0.92). The correlation coefficients between the expression of autophagy-related proteins LC3 II/LC3 I, Beclin1, P62, and CX43 protein were −0.76, −0.81, and 0.75, respectively, indicating that there was a significant negative correlation between LC3 II/LC3 I, Beclin1 protein expression and CX43 protein expression, and a significant positive correlation between P62 protein expression and CX43 protein expression. The correlation coefficients between the expression of autophagy-related proteins LC3II/LC3 I, Beclin1, P62, and the VFT were −0.84, −0.82, and 0.86, respectively, indicating that there was a significant negative correlation between the expression of LC3II/LC3 I, Beclin1 protein and the VFT, and a significant positive correlation between P62 protein expression and the VFT (Figure 1(d)).

### 3.2. Effects of WenXin KeLi on Arrhythmia and Cardiac structure and Function in Rats with MI

**Figure 2:**
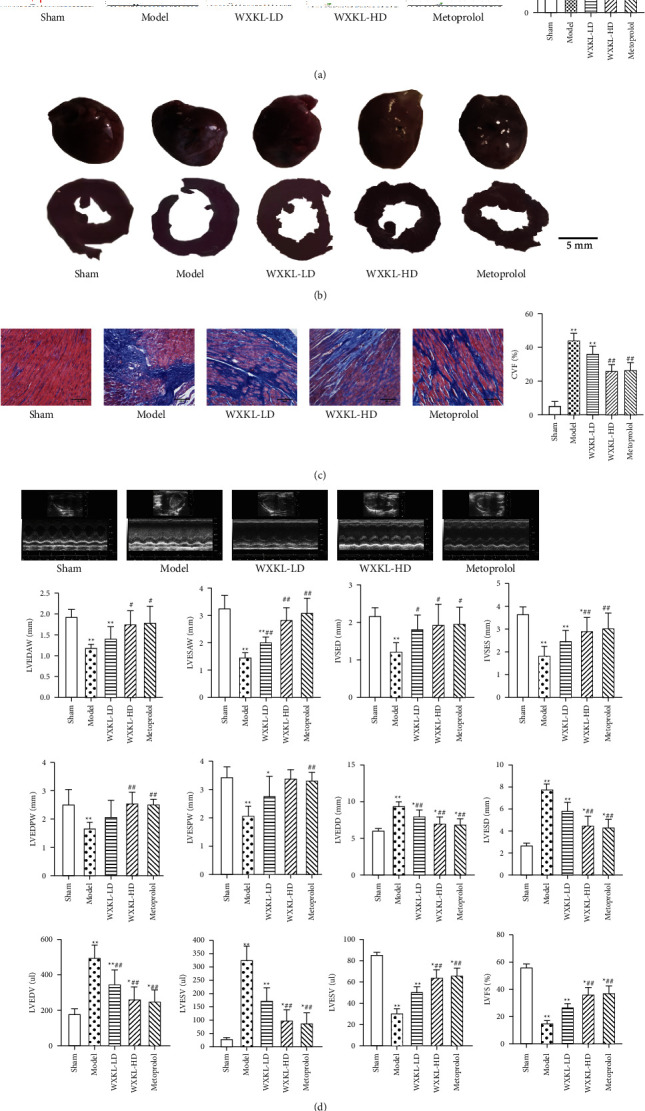
Effects of WenXin KeLi on arrhythmia and cardiac structure and function in rats with MI. (a) VFT of rats was measured by electrical stimulation; (b) gross and section views of rat hearts; (c) Masson staining was used to determine the CVF of rats; (d) cardiac structural and functional changes were determined by cardiac ultrasound. Compared with the sham group, ^*∗*^*P* < 0.05 and ^*∗∗*^*P* < 0.01. Compared with the model group, ^#^*P* < 0.05 and ^##^*P* < 0.01.

Four weeks after the operation, the VFT induced by electrical stimulation in the WXKL-HD group and metoprolol group was significantly higher than that in the model group (Figure 2(a)). Compared with the sham group, the color of the infarcted area of the left ventricle became white, the ventricular wall became thinner and collapsed widely and the ventricular cavity enlarged in the model group. Compared with the model group, the white fibrotic area in the marginal zone of ventricular infarction decreased and the area of the ventricular cavity decreased in each drug group (Figure 2(b)). Masson staining showed that there was a small amount of collagen in the myocardial space of the sham group. Compared with the sham group, the model group had a large amount of collagen deposition in the myocardial space, myocardial fibrosis was obvious, and the myocardial CVF was significantly increased. Compared with the model group, the CVF in the WXKL-LD group, WXKL-HD group, and metoprolol group decreased significantly (Figure 2(c)). The results of ultrasound showed that compared with the model group, LVESAW and IVSED increased, and LVEDD and LVEDV decreased significantly in the WXKL-LD group. In the WXKL-HD group and metoprolol group, LVEDAW, LVESAW, IVSED, IVSES, LVEDPW, LVESPW, LVEF, and LVFS increased significantly, LVEDD, LVESD, LVEDV, and LVESV decreased significantly (Figure 2(d)).

### 3.3. Effect of WenXin KeLi on CX43 Protein Expression

**Figure 3:**
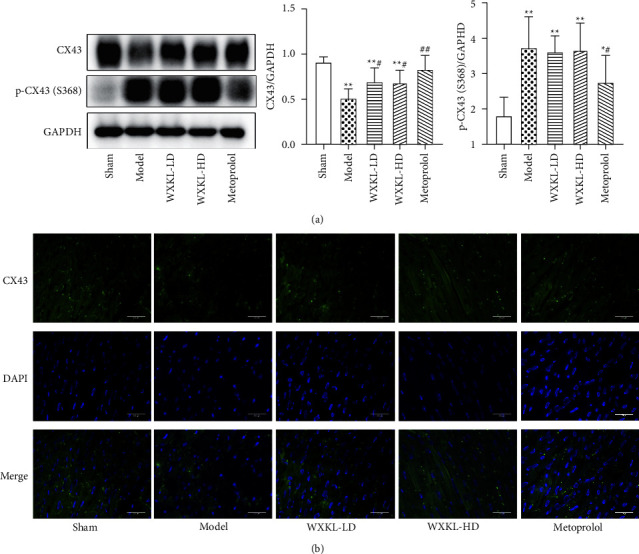
Effect of WenXin KeLi on CX43 protein expression. (a) The expression of CX43 protein in each group was detected by Western Blot; (b) the distribution of CX43 in each group was observed by immunofluorescence. Compared with the sham group, ^*∗*^*P* < 0.05 and ^*∗∗*^*P* < 0.01. Compared with the model group, ^#^*P* < 0.05 and ^##^*P* < 0.01.

Compared with the model group, after four weeks of treatment with WenXin KeLi, the expression of CX43 protein increased while there was no statistical difference in the expression of p-CX43(S368) (Figure 3(a)). The immunofluorescence showed that CX43 in the sham group was mainly distributed in the intercalated disc between myocardial cells, which were arranged neatly in line. The distribution of CX43 in the model group was disordered and the fluorescence intensity decreased. Compared with the model group, the distribution of CX43 in each drug group improved (Figure 3(b)).

### 3.4. Effect of WenXin KeLi on the Expression of Autophagy-Related Protein

**Figure 4:**
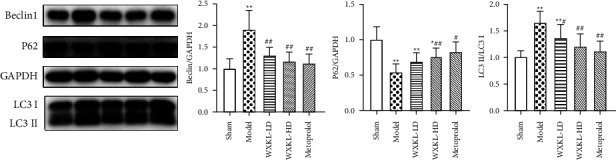
The expression of Beclin1, P62, and LC3II/LC3I protein was detected by Western Blot. Compared with the sham group, ^*∗*^*P* < 0.05 and ^*∗∗*^*P* < 0.01. Compared with the model group, ^#^*P* < 0.05 and ^##^*P* < 0.01.

Compared with the model group, the expression of LC3II/LC3I and Beclin1 protein decreased significantly in each drug group and the expression of P62 protein increased significantly in the WXKL-HD group and metoprolol group (Figure 4).

### 3.5. Effect of WenXin KeLi on PI3K-AKT-mTOR Autophagy Pathway in Rats with MI

**Figure 5:**
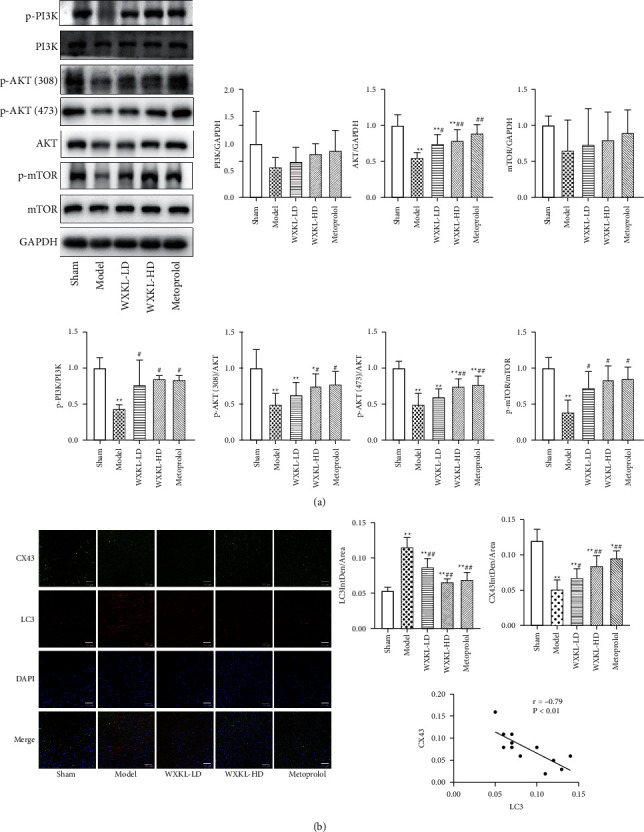
Effect of WenXin KeLi on PI3K-AKT-mTOR autophagy pathway in rats with MI. (a) Protein expression of PI3K-AKT-mTOR pathway in rats of each group was detected by Western Blot; (b) CX43 and LC3 fluorescence intensity were detected by confocal and the correlation analysis of fluorescence intensity between LC3 and CX43. Compared with the sham group, ^*∗*^*P* < 0.05 and ^*∗∗*^*P* < 0.01. Compared with the model group, ^#^*P* < 0.05 and ^##^*P* < 0.01.

Four weeks after the establishment of the MI model, the protein expression of p-PI3K, AKT, p-AKT (473), p-AKT (308), and p-mTOR in the model group were significantly lower than those in the sham group. Compared with the model group, the expression of AKT, p-PI3K, p-AKT (308), p-AKT (473) and p-mTOR increased significantly after four weeks of treatment with WenXin KeLi and Metoprolol (Figure 5(a)). Four weeks after the operation, the laser confocal results showed that the fluorescence intensity of LC3 in the model group was significantly higher than that in the sham group. Compared with the model group, the fluorescence intensity of LC3 decreased significantly. Compared with the sham group, the fluorescence intensity of CX43 decreased significantly in the model group, while that increased significantly in each drug group. The correlation coefficient between LC3 fluorescence intensity and CX43 fluorescence intensity was -0.79, showing a significant negative correlation (Figure 5(b)).

## 4. Discussion

MI is an acute and critical condition of coronary heart disease. Due to the blocking of the blood supply to the myocardium of the coronary artery, local necrosis occurs in a part of the myocardium due to severe persistent ischemia. With the expansion of infarct scope, heart failure or arrhythmia will eventually lead to death [2]. Through the experiment of ventricular fibrillation induced by electrical stimulation, we have observed that the VFT decreased significantly in the model group. The VFT is the minimum current intensity of ventricular fibrillation, which can directly reflect the cardiac electrical stability and ventricular fibrillation sensitivity [14]. The decrease of VFT indicates that the sensitivity of infarcted myocardium to arrhythmia is enhanced. Further exploration of the internal mechanism of arrhythmia after MI is of great significance to improve the prognosis and outcome of the disease.

In the heart, connexin is the basis for maintaining normal communication, electrical conduction, and rhythmic contraction between cardiomyocytes [15]. Connexins form a half-channel in the form of the hexamer, and the two half-channels of adjacent cells are docked to form a gap junction channel, which is a transmembrane channel of electrical and chemical coupling between cardiomyocytes [5, 16]. In this experiment, the expression of CX43 decreased and the phosphorylated expression of the Ser368 site of CX43 increased significantly in the model group after MI. The phosphorylation change of the S368 site could lead to the internalization and degradation of connexin, resulting in the closure of the gap junction channel [17]. The distribution of CX43 also changed from the end-to-end junction located at the leap disc to the side-to-side junction parallel to the long axis of cardiac fibers, and the arrangement of CX43 was disordered or even disappeared, resulting in abnormal conduction of impulses and changes in synchronization and coordination of cardiac electrical activity, eventually resulting in arrhythmia. Correlation analysis showed that there was a significant positive correlation between CX43 and VFT after MI, that is, the expression of CX43 decreased, the VFT decreased, and the risk of arrhythmia increased. Since improving the expression of CX43 can effectively reduce the occurrence of arrhythmia after MI [18]. Therefore, further research on the molecular mechanism of regulating connexin could provide ideas for the prognostic treatment of MI.

In recent years, the role of autophagy in cardiovascular diseases has attracted extensive attention [19]. Adaptive autophagy can maintain cell dynamic balance through degradation and recycling of damaged organelles; however, excessive and persistent autophagy can degrade normal organelles and consume energy, thus inducing cell death [3, 4]. Some studies have shown that atrial fibrillation is associated with impaired cardiac autophagy [20], while other studies have shown that autophagy increases in cardiomyocytes of severe mitral regurgitation with atrial fibrillation [21]. Therefore, the relationship between arrhythmia and autophagy after MI still needs further experimental study. Western Blot results showed that the expression of autophagy markers LC3II/LC3 I and Beclin1 were significantly up-regulated and P62 was downregulated in rats with MI. LC3 is an autophagy marker protein. Its C terminal is cleaved by cysteine protease (ATG4B) to form LC3-I. LC3-I combines with phosphatidylethanolamine (PE) to form LC3-II, which participates in the extension of the autophagy membrane and is related to the formation of the autophagosome. Therefore, when the expression of LC3-II increases, autophagy is activated [22]. Beclin1, as a specific autophagy protein, can regulate autophagosome formation. Thus, the significant up-regulation of Beclin1 gene activity indicates autophagy activation [23]. P62, an autophagy substrate protein with multiple domains, binds to LC3 through its C-terminal LC3 interacting region (LIR) and aggregates the protein to be degraded. During the process of protein accumulation, P62 expression increases, but decreases when the autophagy lysosomal maturation degradation reaction begins [24]. Therefore, the above results showed that the level of autophagy of cardiomyocytes in the model group increased after MI.

The results of correlation analysis between autophagy and CX43 showed that there was a significant negative correlation between Beclin1, LC3II/LC3I, and CX43 expression, and a significant positive correlation between P62 and CX43 expression, indicating that the increase of autophagy level after MI was correlated with the increase of CX43 degradation. Autophagy was found to be involved in the degradation of connexin [25]. The internalization and degradation of CX43 in myocardial tissue of dogs with heart failure can be mediated by the autophagolysosomal pathway [6]. The overactivation of autophagy can mediate increased CX43 degradation and induce increased apoptosis in H9c2 cardiomyocytes [26]. The above studies demonstrated the correlation between enhanced autophagy levels and increased CX43 degradation after MI, while increased CX43 degradation can lead to an increased risk of arrhythmia, which further proved that autophagy was associated with arrhythmia after MI. In this study, the correlation analysis between autophagy and VFT after MI showed that there was a significant negative correlation between cardiomyocyte autophagy-related proteins Beclin1, LC3II/LC3I, and VFT, and a significant positive correlation between P62 expression and VFT, suggesting that the overactivation of myocardial autophagy after MI was related to the increased risk of arrhythmia. There was a correlation between enhanced autophagy and arrhythmia. During myocardial ischemia-reperfusion in mice, the expression levels of autophagy markers Beclin1 and LC3II in the model group with ventricular fibrillation were significantly higher than those in the control group without ventricular fibrillation [27]. Inhibiting the level of autophagy can reduce the incidence of arrhythmia in desmosome hereditary heart disease [28]. The above studies further supported the fact that the activation of autophagy increased the risk of arrhythmia in rats with MI. In summary, the above studies showed that the increased risk of arrhythmia after MI was related to the increased degradation of CX43. The abnormal distribution and quantity of CX43 lead to intercellular conduction dysfunction, which can induce arrhythmia. The activation of autophagy was significantly correlated with the increase of CX43 degradation and the risk of ventricular fibrillation, suggesting that the pathological basis of malignant arrhythmia after MI was related to the excessive stress of autophagy and the increase of CX43 degradation.

WenXin KeLi is a TCM preparation for the treatment of arrhythmia in the clinic, which has significant efficacy in alleviating arrhythmia symptoms after MI and improving patients' survival quality [29]. In this experiment, we have observed that compared with the model group, the scope of MI in the WenXin KeLi group was significantly reduced. Masson staining showed that the scope of myocardial fibrosis was significantly reduced, the disordered arrangement of cardiomyocytes was improved, and the pathological changes in the heart were gradually reduced. Ultrasound detection results showed that after WenXin KeLi treatment, the structure and function of the heart were significantly improved, the ventricular wall was thickened, myocardial contractility was enhanced, cardiac ejection fraction and stroke output were increased, and the VFT of rats was significantly higher than that in model group. The results showed that WenXin KeLi could improve the cardiac structure and function of rats after MI, reduced the risk of arrhythmia complications after MI, and played a role in cardiac protection, which laid a foundation for further research on the molecular mechanism of WenXin KeLi in protecting the heart.

However, whether the pharmacological effect of WenXin KeLi on improving the prognosis of MI was related to the regulation of cardiac autophagy level and Cx43 expression still lacks of experimental evidence. This study further investigated the effects of Wenxin KeLi on autophagy and CX43 expression after MI. The Western Blot results showed that compared with the model group, the expression of autophagy positively related proteins Beclin1 and LC3II/LC3I decreased and the expression of autophagy negative regulator P62 increased in the WenXin KeLi group, indicating that Wenxin KeLi could significantly reduce the level of overactivated autophagy after MI. WenXin KeLi can regulate autophagy and inhibit cardiomyocyte hypertrophy induced by angiotensin II [30]. Ginsenoside Rb1 could inhibit cardiomyocyte autophagy and reduce myocardial ischemia-reperfusion injury [31]. Ginsenoside Rb1 was the active chemical component in WenXin KeLi, which further proved the regulatory effect of WenXin KeLi on the overactivated autophagy level after MI. Our study observed that in the WenXin KeLi group, the expression of CX43 was increased, the distribution was improved, and the communication connection, electrical conduction, and rhythmic contraction between cardiomyocytes gradually recovered, and the pathological changes in cardiac structure and function were improved after MI. WenXin KeLi also could protect MI by improving CX43 expression through miR-1 [32]. The chemical component ginsenoside Rg1, a chemical component in WenXin KeLi, could regulate the biosynthesis and degradation of CX43 [33], indicating that WenXin KeLi could improve the expression and distribution disorder of CX43. The above results showed that 4 weeks after MI, the autophagy of the model group was overactivated, the degradation of CX43 was increased, and the risk of arrhythmia was increased. WenXin KeLi could reduce the overactivated autophagy level, improve the expression of CX43 and play an antiarrhythmic role.

The PI3K-AKT-mTOR signaling pathway is a classical pathway that regulates the autophagy mechanism [10]. PI3K includes three subtypes: type I, II, and III. Type I PI3K activation is an important segment in regulating the mTOR autophagy pathway. AKT is the main downstream target of PI3K. The activation of AKT lies in the phosphorylation of its threonine 308 and serine 473 sites. The activated AKT is transferred to the cytoplasm or nucleus to regulate the downstream target protein mTOR [34]. mTOR, as the main inhibitory signal in the process of autophagy, is positively regulated by the PI3K-AKT pathway [35]. Studies have shown that activation of the PI3K-AKT-mTOR signaling pathway could reduce excessive autophagy and myocardial injury of myocardial cells after ischemia-reperfusion [31]. In this experiment, compared with the sham group, the expression of AKT in MI rats decreased significantly, PI3K, AKT and mTOR dephosphorylated and inactivated, and the expression of LC3II increased significantly. The confocal results showed that there was a significant negative correlation between LC3 and CX43 in the model group, indicating that the PI3K-AKT-mTOR signal pathway was inhibited, autophagy was overactivated, and the expression of connexin was decreased after MI. These alterations constituted an intrinsic pathological mechanism of the signal transduction disorder between cardiomyocytes and the increased risk of arrhythmia after MI, which aggravated the disease progression after MI. The overall animal research results were consistent with the observation that the PI3K-AKT-mTOR pathway regulated autophagy activity and downregulated CX43 in H9c2 cardiomyocytes [36]. Compared with the model group, after 4 weeks of WenXin KeLi intervention, the PI3K-AKT-mTOR signal pathway was activated and the phosphorylation expression of PI3K, threonine 308, and serine 473 sites of AKT, as well as mTOR, were significantly increased, while the expression of LC3II was reduced, the autophagy overactivation state was inhibited, and CX43 expression was increased. In conclusion, the results indicated that WenXin KeLi could activate the PI3K-AKT-mTOR signal pathway, inhibit excessive autophagy in cardiomyocytes, improve connexin expression and distribution, then improve the prognosis of MI and eventually exert the cardioprotective molecular mechanism.

## 5. Conclusion

**Figure 6:**
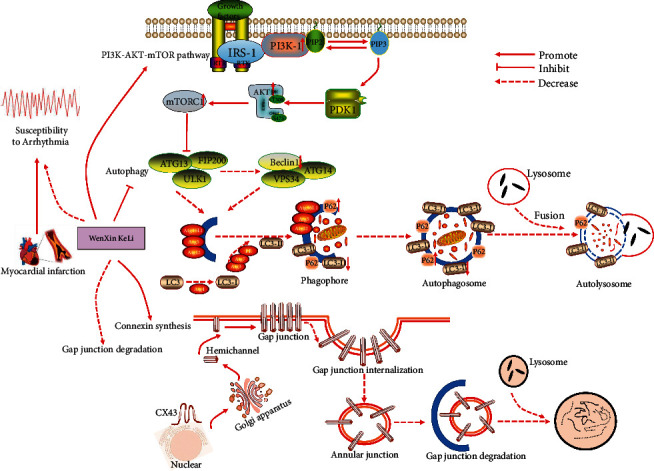
The increased risk of arrhythmia after MI was related to the overactivation of autophagy after MI. WenXin KeLi could activate the PI3K-AKT-mTOR signal pathway, up-regulate the phosphorylation expression of PI3K, AKT, and mTOR proteins, inhibit the level of cardiac autophagy, reduce the expression of Beclin1 and LC3-II proteins, increase the expression of the P62 protein, and reduce the risk of arrhythmia after MI. The increased risk of arrhythmia after MI was related to the decreased expression of CX43 after MI. The internalization and degradation of CX43 increased after MI. WenXin KeLi could significantly increase the expression of CX43, reduce gap junction degradation and reduce the risk of arrhythmia after MI.

Our study confirmed at the basic experimental level that the increased risk of arrhythmia after MI was closely related to the hyperactivation of autophagy and the decreased expression of CX43 after MI. It revealed that the cardioprotective mechanism of WenXin KeLi in improving cardiac structure and function and reduced the risk of arrhythmia in rats after MI, which was related to the activation of the PI3K- AKT-mTOR signal pathway and the improvement of cardiac autophagy and CX43 expression in rats after MI (Figure 6).

## Data Availability

The data used to support the findings of this study are available from the corresponding author upon request.
